# Serum Sclerostin and Its Association with Bone Turnover Marker in Metabolic Bone Diseases

**DOI:** 10.1155/2022/7902046

**Published:** 2022-09-10

**Authors:** Lihui Chen, Gao Gao, Li Shen, Hua Yue, Ge Zhang, Zhenlin Zhang

**Affiliations:** ^1^Department of Osteoporosis and Bone Disease, Shanghai Clinical Research Center of Bone Disease, Shanghai Jiao Tong University Affiliated Sixth People's Hospital, Shanghai 200233, China; ^2^Medical Center, Shanghai Jiao Tong University Affiliated Sixth People's Hospital, Shanghai 200233, China; ^3^Clinical Research Center, Shanghai Jiao Tong University Affiliated Sixth People's Hospital, Shanghai 200233, China; ^4^Law Sau Fai Institute for Advancing Translational Medicine in Bone and Joint Diseases, School of Chinese Medicine, Hong Kong Baptist University, Kowloon Tsai, Hong Kong

## Abstract

Sclerostin is a secreted inhibitor of Wnt/*β*-catenin signaling that is mainly produced by osteocytes and is an important regulator of bone remodeling. Some studies have evaluated serum sclerostin levels in metabolic bone diseases, but the results have been contradictory. The profile of serum sclerostin levels in patients with osteogenesis imperfecta (OI), X-linked hypophosphatemia (XLH), and Paget's disease of bone (PDB) was obtained to determine their association with bone turnover marker. Serum sclerostin levels, biochemical parameters, and the bone turnover marker, *β*-CrossLaps of type 1 collagen containing cross-linked C-telopeptide (*β*-CTX), were measured in 278 individuals, comprising 71 patients with OI, 51 patients with XLH, 17 patients with PDB, and 139 age- and sex-matched healthy controls. A correlation analysis was performed between sclerostin and *β*-CTX concentration. The univariate logistic regression analysis was used to analyze factors associated with OI, XLH, and PDB. Patients with PDB (11 male 6 female), aged 44.47 ± 14.75 years; XLH (17 male, 34 female), aged 19.29 ± 15.65 years; and OI (43 male, 28 female), aged 19.57 ± 16.45 years, had higher sclerostin level than age- and sex-matched healthy controls [median(interquartile range): 291.60 (153.42, 357.35) vs. 38.00 (27.06, 68.52) pmol/L, 163.40 (125.10, 238.20) vs. 31.13 (20.37, 45.84) pmol/L, and 130.50 (96.12, 160.80) vs. 119.00 (98.89, 194.80) pmol/L, respectively; *P* < 0.001]. Patients with PDB had the highest level of serum sclerostin, followed by those with XLH and OI (*P* < 0.05). Sclerostin was positively correlated with *β*-CTX in OI and XLH (*r* = 0.541 and *r* = 0.661, respectively; *P* < 0.001). Higher *β*-CTX and sclerostin levels were associated with a higher risk of OI, XLH, and PBD. Sclerostin may be a biomarker of OI, XLH, and PDB. Whether sclerostin inhibitors can be used in these patients requires further analysis using additional cohorts.

## 1. Introduction

Sclerostin is a glycoprotein secreted mainly by mature osteocytes. It is an important regulator of bone remodeling by inhibiting Wnt/*β*-catenin signaling [1]. The Wnt/*β*-catenin signaling pathway regulates the maturation and differentiation of osteoblasts and increases the expression of osteoprotegerin (OPG) in osteoblasts and osteocytes to inhibit the production and differentiation of osteoclasts. Mutations in the *SOST* gene (encoding sclerostin) can cause sclerosteosis; van Buchem disease (VBD) results from osteoblast hyperactivity [2]. Monoclonal antibodies against human sclerostin can stimulate bone formation and inhibit bone resorption. Therefore, antisclerostin therapies are used to improve bone strength and mass in osteoporosis [3].

Osteogenesis imperfecta (OI), X-linked hypophosphatemia (XLH), and Paget's disease of bone (PDB) are metabolic bone diseases. Osteogenesis imperfecta (OI) is a heritable disorder characterized by increased bone fragility and low bone mass. The majority of patients with OI have mutations in *COL1A1* and *COL1A2*, the genes encoding type I collagen [4]. Unlike OI, XLH is a rare disorder of phosphorus metabolism caused by mutations in the phosphate-regulating endopeptidase homolog X-linked (*PHEX*) gene, which encodes phosphate-regulating neutral endopeptidase, PHEX, which is predominantly expressed in osteoblasts, osteocytes, and teeth [5]. Because of renal phosphate wasting, serum phosphorus levels in patients with XLH are below the age-related reference range [6]. PDB is a high-turnover bone disorder that primarily occurs in middle-aged or older individuals. Enhanced bone resorption due to the increased activity of osteoclasts recruits a large number of osteoblasts to resorption sites, resulting in a large amount of new bone matrix [7].

Since sclerostin plays an important role in both bone formation and resorption, sclerostin-mediated signal transduction changes may play a role in the pathogenesis of metabolic bone diseases. Some studies have evaluated serum sclerostin levels in these metabolic bone diseases, but the results have been contradictory. Palomo et al. [8] reported that sclerostin levels in 76 patients with OI were similar to those in healthy controls; the same finding was observed by Brunetti et al. [9] in 18 patients with OI. Studies by Nicol et al. [10] and Kocijan et al. [11] showed that patients with OI had lower serum sclerostin levels than age- and sex-matched controls (sample sizes: *n* = 66 and 27, respectively). Yavropoulou et al. [12] studied 88 patients with PDB and found that they had substantially higher circulating sclerostin levels than the healthy controls, whereas no difference was observed between 40 patients with PDB and 40 healthy controls in another study [13].

To further understand the potential role of sclerostin in bone metabolism and the serum sclerostin levels in different metabolic diseases, serum sclerostin levels were measured in patients with OI, XLH, and PDB, and the correlation between serum sclerostin levels and *β*-CrossLaps of type 1 collagen containing cross-linked C-telopeptide (*β*-CTX) was analyzed.

## 2. Materials and Methods

### 2.1. Study Subjects

A total of 278 individuals were selected from the database of Shanghai Clinical Research Center of Bone Diseases, which was established by the Department of Osteoporosis and Bone Diseases at Shanghai Jiao Tong University Affiliated Sixth People's Hospital in 2010. These individuals included 71 patients who have clinical features of OI and mutations of *COL1A1*and *COL1A2* gene (43 male, 28 female, aged 1-62 years) [14], 51 XLH patients identified with *PHEX* gene mutation (17 male and 34 female, aged 1.3-62 years) [6], 17 patients with radiological and scintigraphic evidence of PDB (11 male and 6 female, aged 26-82 years), and 139 age- and sex-matched healthy controls without clinically significant abnormalities in laboratory results who underwent routine physical examination(71 male and 68 female, aged 15-82 years).

Individuals with impaired renal function, impaired liver function, or the presence of diseases known to increase bone turnover other than OI, XLH, and PDB and those who take bisphosphonates, denosumab, and phosphate were excluded. This study was approved by the Ethics Committee of Shanghai Jiao Tong University Affiliated Sixth People's Hospital, and informed consent was obtained from all participants or from participants' parents if they were under 18 years old.

### 2.2. Biochemical Measurements

Fasting blood samples were collected by inert separation gel vacuum procoagulant collective tube for biochemical tests. Serum phosphorus, calcium, serum alkaline phosphatase (ALP), serum creatinine (Cr), intact parathyroid hormone (PTH), 25-hydroxyvitamin D (25OHD), *β*-CTX, and serum osteocalcin in the form of an N-terminal midmolecule fragment (OC) were measured.

Serum phosphorus, calcium, and ALP levels were measured using a Hitachi 7,600-020 automatic biochemistry analyzer. Serum PTH, 25OHD, *β*-CTX, and OC concentrations were assessed using an automated Roche electrochemiluminescence system (E170; Roche Diagnostic GmbH, Mannheim, Germany) [15]. The performance characteristics of the methods for all measured biomarkers are shown in Table 1.

### 2.3. Serum Sclerostin Measurement

Serum samples were collected to measure sclerostin levels at each patient's first visit to Shanghai Jiao Tong University Affiliated Sixth People's Hospital from 2010 to 2020. All the serum samples were adequately stored at -80°C and unthawed before measured for sclerostin. Serum sclerostin was measured by enzyme-linked immunosorbent assay (ELISA) using polyclonal goat anti-human sclerostin as the capture antibody, biotin-labeled monoclonal mouse anti-human sclerostin as the detection antibody, and horseradish peroxidase-streptavidin and tetramethylbenzidine for the chromogenic reaction (Biomedica Medizinproduckte GmbH and Co.,KG). The detection limit of the assay is 320 pmol/L, samples with values above 320 pmol/L can be diluted. The intra- and interassay precision are ≤1% and ≤5%, respectively. The limit of detection (LOD) and lower limit of quantification (LLOQ) are 1.9 pmol/L and 1.3 pmol/L, respectively. The median value of apparently healthy individuals in serum is 61.5 pmol/L.

### 2.4. Statistical Analyses

SPSS 26.0 was used for the statistical analysis. The continuous variables that conformed to the normal distribution were expressed as mean ± standard deviation (SD), and the *t*-test was used for comparison between groups. The continuous variables that did not conform to the normal distribution were expressed as median (interquartile range), and independent comparisons were performed using the independent sample Mann–Whitney *U* test. The categorical variables were expressed as frequencies, and the chi-square test was used for comparison between groups. The Spearman correlation analysis was performed between sclerostin and other factors. The univariate logistic regression analysis was used to analyze the factors associated with OI, XLH, and PDB. A value of *P* < 0.05 was defined as significant.

## 3. Results

The basic characters of the 278 individuals were presented in Tables 2–4. No differences were observed in age, ratio males/females, and BMI between patients and the control groups. Compared with age- and sex-matched control participants, patients with OI, XLH, and PDB had higher serum *β*-CTX and sclerostin levels (Tables 2–4).

**Figure 1:**
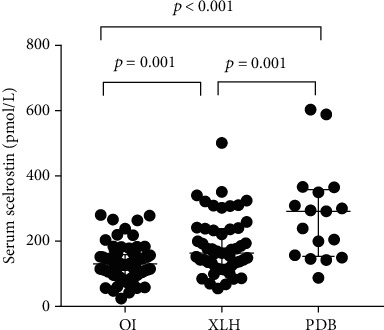
Individual values of serum sclerostin levels in the study group. OI: osteogenesis imperfecta; XLH: X-linked hypophosphatemia; PDB: Paget's disease of bone.

Among the three groups, PDB patients had a median sclerostin level of 291.60 pmol/L (interquartile range: 153.42-357.35 pmol/L), XLH patients had a lower median sclerostin level of 163.40 pmol/L (interquartile range: 125.10-238.20 pmol/L), and OI patients had a lowest median sclerostin level of 130.50 pmol/L (interquartile range: 96.12-160.80 pmol/L) (Table 5 and Figure 1). In XLH, serum sclerostin levels were significantly higher in male patients compared with female patients [236.00 (163.26-309.70) vs. 142.98 (113.75-179.28) pmol/L, *P* = 0.003]. There were no differences regarding serum sclerostin levels between genders in OI and PDB [male vs. female, 127.30 (95.68-151.80) vs. 130.50 (101.84-176.65) pmol/L and 294.00 (199.68-364.80) vs. 218.80 (128.42-316.60) pmol/L, respectively, all *P* > 0.05]. *β*-CTX levels were significantly higher in XLH and PDB than that in OI (*P* = 0.001 and *P* = 0.017, respectively), no significant differences were observed between XLH and PDB. XLH patients had a higher PTH level than OI and PDB (*P* < 0.001 and *P* = 0.002, respectively). 25-OHD and OC level were similar in OI, XLH, and PDB.

**Figure 2:**
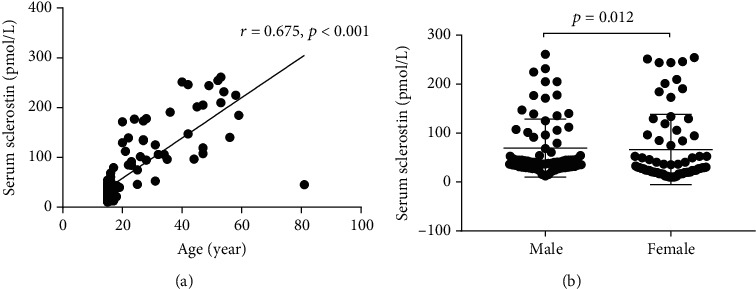
(a) Serum sclerostin concentration and its correlation with age in all healthy controls. (b) Comparison of serum sclerostin level by gender.

In total healthy controls, the median sclerostin level was 43.1 pmol/L (interquartile range: 27.85-132.63 pmol/L), and sclerostin has a positive correlation with age (r =0.675, *P* <0.001). The level of sclerostin in male individuals was significantly higher than that in female individuals [42.73 (31.77,96.28) vs. 29.98 (19.98,91.99) pmol/L, *P* = 0.012] (Figure 2).

The patients and age- and sex-matched healthy controls were divided as three groups, the OI group (71 OI patients and 71 age- and sex-matched controls), the XLH group (51 XLH patients and 51 age- and sex-matched controls) and the PDB group (17 PDB patients and 17 age- and sex-matched controls). Correlation analysis was performed between sclerostin and *β*-CTX in three groups (Table 6). For the OI group, sclerostin was positively correlated with *β*-CTX (*r* = 0.541, *P* < 0.001). For serum sclerostin in the XLH group, the positively correlations with *β*-CTX (*r* = 0.661, *P* < 0.001) were found. For the PDB group, no significantly correlation between sclerostin and *β*-CTX was observed.

Next, the univariate logistic regression analysis was used to investigate the association between *β*-CTX and sclerostin and OI, XLH, and PBD (Table 7). The univariate logistic regression analysis indicated that a 100 ng/L increase in *β*-CTX was associated with an odds ratio (OR) of 1.344, 1.497, and 1.824 for having OI (OR = 1.344, 95% confidence interval, 1.138 to 1.588), XLH (OR = 1.497, 95% confidence interval, 1.215 to 1.845) and PDB (OR = 1.824, 95% confidence interval, 1.148 to 2.900). Also, a 50 pmol/L increase in sclerostin was associated with an OR of 2.602, 4.500, and 2.166 for having OI (OR = 2.602, 95% CI, 1.833 to 3.693), XLH (OR = 4.500, 95% CI, 2.653 to 7.631) and PDB (OR = 2.166, 95% CI, 1.227 to 3.826). It is suggested that higher levels of *β*-CTX and sclerostin were associated with the higher risk of OI, XLH, and PBD.

## 4. Discussion

In our investigation, we examined the levels of circulating sclerostin in patients with OI, XLH, and PDB and in age- and sex-matched healthy control individuals. We discovered that the greatest amounts of serum sclerostin were found in PDB patients, which were followed by XLH and OI patients. Healthy control individuals had the lowest levels. Sclerostin was identified as a risk factor for OI, XLH, and PDB by the univariate logistic regression analysis.

As described in the introduction section, the study findings on the sclerostin levels in these metabolic diseases were contradictory. To date, only four studies have reported sclerostin levels in individuals with OI (sample sizes: *n* = 76, 18, 66, and 27) [8–11]. Three studies have reported sclerostin levels in individuals with XLH (sample sizes: *n* = 30, 27, and 24) [8, 16, 17], and three studies have reported sclerostin levels in individuals with PDB (sample sizes: *n* = 88, 40, and 57) [12, 13, 18]. We found that the sclerostin levels were higher in OI patients than in control individuals, but Palomo et al. [8] and Brunetti et al. [9] reported that the sclerostin levels of OI patients were similar to those of healthy control individuals (sample sizes: *n* = 76 and 18, respectively). Two other studies by Nicol et al. [10] and Kocijan et al. [11] showed that individuals with OI had lower serum sclerostin levels than age- and sex-matched control individuals (sample sizes: *n* = 66 and 27, respectively). Some studies have demonstrated that there may actually be an increase in the numbers or density of osteocytes in OI patients [19, 20], which indicates that high sclerostin levels are reasonable. OI patients may experience limitations in activity due to fractures and musculoskeletal complications, which can lead to increased sclerostin levels [21]. The inconsistent results from these studies may be related to the age of the participants. A study showed that sclerostin levels were positively correlated with bone age prior to pubertal onset and negatively correlated with bone age after puberty in children aged 6-21 years [22]. Regulation of sclerostin may vary during growth, and the wide variation in levels observed in children during development may make it more difficult to detect differences between control individuals and individuals with OI [23].

We demonstrated that XLH patients had higher sclerostin levels than control individuals, which were consistent with previous studies [8, 16, 17]. A study found that sclerostin expression was increased in the femoral bone in an XLH mouse model (Hyp mouse) [24]. Additionally, XLH patients typically have high bone mass [25], which may result in increased circulating sclerostin levels.

Regarding PDB, our finding is in accordance with Yavropoulou et al. [12] who found that sclerostin levels were higher in PDB patients than in control individuals. The mechanism responsible for the increase in sclerostin in PDB is unknown. A previous study showed that sclerostin may have a catabolic action through the promotion of osteoclast formation and activity by osteocytes in a RANKL-dependent manner [26], since sclerostin has an effect both on bone formation and resorption. In PDB, the increased sclerostin level could be caused by accelerated bone formation, or the increased sclerostin level could cause enhanced bone resorption. Further studies will be necessary to elucidate the role of sclerostin in PDB. The different results in the other two studies [13, 18] may be because of population differences and different sclerostin assays.

In our study, the sclerostin level was positively correlated with *β*-CTX levels in OI and XLH patients. In OI, the relationship between sclerostin and *β*-CTX is controversial. Palomo et al. [8] and Nicol et al. [10] showed that there was no significant correlation between *β*-CTX levels and circulating sclerostin in 76 children and young adult patients and 66 adult patients, respectively. Another study showed a negative correlation in 27 adult patients [11]. In XLH patients, sclerostin levels were not correlated with *β*-CTX levels in a study of 27 adult patients conducted by Hansen et al. [16]. The discordant results from these studies may be related to the age of the participants. Our previous study showed that serum sclerostin levels were negatively correlated with *β*-CTX levels in Chinese community-dwelling elderly individuals and positively correlated in adolescents [27]. The same findings were also observed in white postmenopausal women, where serum sclerostin levels were negatively correlated with *β*-CTX levels [28]. The existing study results showed no significant correlations between sclerostin levels and CTX levels in PDB patients [12, 13]. In PDB, bone formation and resorption are both increased, and the exact mechanisms regulating sclerostin remain unclear.

Our findings also showed that sclerostin and *β*-CTX levels were risk factors for OI, XLH, and PDB. These results suggest that both altered sclerostin-mediated signaling and increased bone resorption have a role in the pathogenesis of OI, XLH, and PDB. Bisphosphonates, which act by inhibiting osteoclast activity and bone resorption, are now the mainstay of pharmacologic treatment in patients with OI and PDB [29, 30]. Thus, sclerostin may also be a promising therapeutic target for these diseases. Trials have been initiated to explore the influence of sclerostin inhibitors on these diseases, with results showing that anti-sclerostin antibodies could improve bone metabolism. Trials in animal models of OI [31, 32] have shown that anti-sclerostin antibodies were able to improve bone strength and microarchitecture and reduce bone fractures. An anti-sclerostin antibody (BPS804) was thereafter tested in clinical trials [33], and improvements were observed. In the XLH mouse model (Hyp mouse), sclerostin antibody treatment increased bone mass and normalized the circulating phosphate levels [34, 35]. Yu et al. [36] summarized the drug discovery of sclerostin inhibitors. They suggest that sclerostin may be a promising therapeutic target for metabolic diseases and that sclerostin inhibitors (monoclonal antibodies, aptamers, and small-molecule inhibitors) could probably be used for patients in the future. However, there were no related trials in PDB patients or mouse models, perhaps because there is evidence that the increased bone formation was secondary to excessive bone resorption [37]. While in the initial state of the onset of PDB, it is possible that the increased bone formation could stimulate bone resorption. Further studies should be conducted in the future, and perhaps sclerostin inhibitors could be used for the treatment of PDB.

Some limitations of this study should be discussed. The healthy control individuals in our study did not undergo the OC or P1NP test, and we did not analyze the correlations between bone formation markers and sclerostin. In addition, because PDB is very rare in China, the sample size of PDB patients in our study was small, although we obtained results that are consistent with previous research.

In conclusion, we demonstrate increased serum sclerostin levels in three metabolic bone diseases. Diseases with a high rate of bone turnover had high sclerostin levels, and PDB patients had the highest level of serum sclerostin, followed by XLH and OI. Sclerostin plays an important role in bone metabolism, and the inhibition of sclerostin could be an option for improving metabolic bone diseases.

## Figures and Tables

**Table 1:** The performance characteristics of the methods for all measured biomarkers.

	Precision	Sensitivity	Reference intervals
PTH	Intra-assay ≤ 2.0%, inter-assay ≤ 3.4%	LOD: 1.20 pg/mL	15–65 pg/mL
25OHD	Intra-assay ≤ 6.8%, inter-assay ≤ 13.1%	LOB: 2 ng/ml; LOD: 3 ng/ml; LOQ: 5 ng/ml	> 20 ng/ml
Pi	Intra-assay ≤ 4%, inter-assay < 6%	Δ*A*: (1.3 mmol/L): ≥ 0.0530	0.8–1.6 mmol/L
ALP	Intra-assay ≤ 3.4%, inter-assay ≤ 4.1%	Δ*A*/min: (120 U/L): 0.020–0.100	15–112 U/L
*β*-CTX	Intra-assay ≤ 4.7%, inter-assay ≤ 5.7%	LOD: 10 ng/L	278–540 ng/L[38]
OC	Intra-assay ≤ 1.3%, inter-assay ≤ 2.3%	LOD: < 0.50 ng/mL	13.07–27.68 ng/mL[38]
Cr	Intra-assay ≤ 5.0%, inter-assay ≤ 10.0%	*Δ*A: (100.0 *μ* Mol/L): 0.003-0.040	53.0–115.0 *μ* Mol/L
Ca	Intra-assay ≤ 3%, inter-assay ≤ 5%	$\Delta \underset{¯}{A}$ : (2.5 mmol/L): ≥ 0.0900	2.08-2.60 mmol/L
Sclerostin	Intra-assay ≤ 1%, inter-assay ≤ 5%	LOD: (0 pmol/L +3 SD):1.9 pmol/L; LLOQ: 1.3 pmol/L	Median serum: 61.5 pmol/L

PTH: parathyroid hormone; 25OHD: 25-hydroxyvitamin D; Pi: serum phosphate; ALP: serum alkaline phosphatase; *β*-CTX: *β*-CrossLaps of type 1 collagen containing cross-linked C-telopeptide; OC: serum osteocalcin in the form of an N-terminal midmolecule fragment; Cr: serum creatinine; Ca: serum total calcium; LOD: limit of detection; LOB: limit of blank; LOQ: limit of quantitation; LLOQ: lower limit of quantitation; Δ*A*: absorbance difference.

**Table 2:** Basic characters of OI and the control group.

	Control (*n* = 71)	OI (*n* = 71)	P
Age (year)	19.53 ± 8.84	19.57 ± 16.45	—
Sex (male/female)	43/28	43/28	—
BMI (kg/m^2^)	20.98 ± 3.11	19.90 ± 4.76	0.146
*β*-CTX (ng/L)	435.00 (300.00, 640.00)	793.10 (433.05, 1140.00)	0.001
PTH (pg/mL)	39.90 (30.88, 60.70)	27.62 (20.27, 45.18)	0.003
25OHD (ng/mL)	18.43 ± 5.73	23.18 ± 12.52	0.057
Sclerostin (pmol/L)	38.00 (27.06, 68.52)	130.50 (96.12, 160.80)	< 0.001

BMI: body mass index; *β*-CTX: *β*-CrossLaps of type 1 collagen containing cross-linked C-telopeptide; PTH: parathyroid hormone; 25OHD: 25-hydroxyvitamin D; OI: osteogenesis imperfecta. Normally distributed data are presented as the mean ± standard deviation (SD), nonnormally distributed data are presented as the median (interquartile range).

**Table 3:** Basic characters of XLH and the control group.

	Control(*n* = 51)	XLH(*n* = 51)	*P*
Age (year)	19.29 ± 9.22	19.29 ± 15.65	—
Sex (male/female)	17/34	17/34	—
BMI (kg/m^2^)	21.36 ± 2.97	23.12 ± 6.13	0.114
*β*-CTX (ng/L)	320.00 (210.00, 485.00)	1616.00 (692.80, 2518.00)	< 0.001
PTH (pg/mL)	35.20 (29.10, 43.70)	67.82 (56.72, 86.28)	< 0.001
25OHD (ng/mL)	17.40 ± 5.81	23.75 ± 13.74	0.009
Sclerostin (pmol/L)	31.13 (20.37, 45.84)	163.40 (125.10, 238.20)	< 0.001

BMI: body mass index; *β*-CTX: *β*-CrossLaps of type 1 collagen containing cross-linked C-telopeptide; PTH: parathyroid hormone; 25OHD: 25-hydroxyvitamin D; XLH: X-linked hypophosphatemia. Normally distributed data are presented as the mean ± standard deviation (SD), nonnormally distributed data are presented as the median (interquartile range).

**Table 4:** Basic characters of PDB and the control group.

	Control(*n* = 17)	PDB(*n* = 17)	*P*
Age (year)	44.47 ± 14.75	44.47 ± 14.75	—
Sex (male/female)	11/6	11/6	—
BMI (kg/m^2^)	22.53 ± 2.45	24.30 ± 2.47	0.064
*β*-CTX (ng/L)	235.00 (182.50, 380.00)	1195 (643.73, 2733.25)	< 0.001
PTH (pg/mL)	—	40.43 (34.70, 59.81)	—
25OHD (ng/mL)	18.38 ± 4.37	22.57 ± 17.54	0.308
Sclerostin (pmol/L)	119.00 (98.89, 194.80)	291.60 (153.42, 357.35)	< 0.001

BMI: body mass index; *β*-CTX: *β*-CrossLaps of type 1 collagen containing cross-linked C-telopeptide; PTH: parathyroid hormone; 25OHD: 25-hydroxyvitamin D; PDB: Paget's disease of bone. Normally distributed data are presented as the mean ± standard deviation (SD), nonnormally distributed data are presented as the median (interquartile range).

**Table 5:** Clinical characteristics of the 139 patients.

	OI (*n* = 71)	XLH (*n* = 51)	PDB (*n* = 17)
*β*-CTX (ng/L)	793.10 (433.05, 1140.00)	1616.00 (692.80, 2518.00)	1195 (643.73, 2733.25)
PTH (pg/mL)	27.62 (20.27, 45.18)	67.82 (56.72, 86.28)	40.43 (34.70, 59.81)
25OHD (ng/mL)	23.18 ± 12.52	23.75 ± 13.74	22.57 ± 10.13
OC (ng/mL)	62.31 (37.16, 111.96)	66.37 (22.63, 98.18)	58.10 (20.48, 140.98)
Ca (mmol/L)	2.49 ± 0.11	2.35 ± 0.12	2.34 ± 0.10
Pi (mmol/L)	1.43 ± 0.31	0.72 ± 0.19	1.17 ± 0.22
ALP (U/L)	228.00 (129.75, 343.25)	307.50 (110.50, 567.00)	293.00 (167.00, 710.50)
Cr (*μ*mol/L)	41.50 ± 15.51	31.97 ± 14.87	53.54 ± 14.40
Sclerostin (pmol/L)	130.50 (96.12, 160.80)	163.40 (125.10, 238.20)	291.60 (153.42, 357.35)

BMI: body mass index; *β*-CTX: *β*-CrossLaps of type 1 collagen containing cross-linked C-telopeptide; PTH: parathyroid hormone; 25OHD: 25-hydroxyvitamin D; OC: serum osteocalcin in the form of an N-terminal midmolecule fragment; Ca: serum total calcium; Pi: serum phosphate; ALP: serum alkaline phosphatase; Cr: serum creatinine; OI: osteogenesis imperfecta; XLH: X-linked hypophosphatemia; PDB: Paget's disease of bone. Normally distributed data are presented as the mean ± standard deviation (SD), nonnormally distributed data are presented as the median (interquartile range).

**Table 6:** Correlation analysis of serum sclerostin and *β*-CTX in all individuals.

	OI (*n* = 142)		XLH (*n* = 102)		PDB (*n* = 34)	
	r	p	r	P	r	p
Age (year)	0.153	0.069	0.123	0.217	0.502	0.002
Sex (male/female)	0.003	0.973	-0.192	0.053	-0.176	0.320
BMI (kg/m^2^)	-0.067	0.457	0.083	0.447	0.518	0.004
*β*-CTX (ng/L)	0.541	< 0.001	0.661	< 0.001	0.213	0.243

BMI: body mass index; *β*-CTX: *β*-CrossLaps of type 1 collagen containing cross-linked C-telopeptide; OI: osteogenesis imperfecta; XLH: X-linked hypophosphatemia; PDB: Paget's disease of bone.

**Table 7:** Univariate logistic regression analysis to explore the relationship between *β*-CTX and sclerostin and OI, XLH, and PBD.

	OI		XLH		PDB	
	OR (95% CI)	*P*	OR (95% CI)	*P*	OR (95% CI)	*P*
*β*-CTX (per 100 ng/L)	1.344 (1.138,1.588)	< 0.001	1.497 (1.215,1.845)	< 0.001	1.824 (1.148, 2.900)	0.011
Sclerostin (per 50 pmol/L)	2.602 (1.833,3.693)	< 0.001	4.500 (2.653,7.631)	< 0.001	2.166 (1.227, 3.826)	0.008

*β*-CTX: *β*-CrossLaps of type 1 collagen containing cross-linked C-telopeptide; OI: osteogenesis imperfecta; XLH: X-linked hypophosphatemia; PDB: Paget's disease of bone; OR: odds ratio; CI: confidence interval.

## Data Availability

The data used to support the findings of this study are available from the corresponding author upon request.
